# Novel Stochastic Sensors Based on Phthalocyanine Complexes for the Detection of C-NP, IL-6, and CRP in Cardiovascular Diseases

**DOI:** 10.3390/life16020339

**Published:** 2026-02-15

**Authors:** Ruxandra-Maria Ilie-Mihai, Raluca-Ioana Stefan-van Staden

**Affiliations:** Laboratory of Electrochemistry and PATLAB, National Institute of Research for Electrochemistry and Condensed Matter, 202 Splaiul Independentei Str., 060021 Bucharest, Romania; i.ruxandra04@yahoo.com

**Keywords:** cardiovascular disease, stochastic sensor, interleukin-6, C-type natriuretic peptide, C-reactive protein

## Abstract

The severity of cardiovascular disease is linked to C-reactive protein, interleukin 6, and C-type natriuretic peptide levels, stressing the need for a sensitive sensor that can detect these biomarkers at ultralow levels in real time. Whole blood samples from confirmed cardiovascular patients were analyzed for C-type natriuretic peptide, C-reactive protein, and interleukin 6 using three stochastic sensors. These sensors were designed using carbon paste matrices decorated with Ag nanoparticles (AgNPs), on which different phthalocyanines were physically immobilized. The sensors exhibited exceptionally low detection limits (1 × 10^−21^ g mL^−1^) and broad linear concentration ranges (1 × 10^−21^ to 1 × 10^−6^ g mL^−1^). The analysis conducted using the Student *t*-test indicated that there is no statistically significant difference between the results obtained from the three stochastic sensors used in the screening tests of whole blood, with ELISA at a confidence level of 99%.

## 1. Introduction

Conditions including coronary artery disease, peripheral artery disease, hypertension, and stroke are all part of the larger category known as cardiovascular diseases (CVDs) [1]. Beyond their obvious physical effects, CVDs have far-reaching psychological and financial consequences for people, families, and healthcare systems around the world [2]. Early detection of cardiovascular disorders and the ability to intervene quickly are both facilitated by routine check-ups with healthcare providers.

Cardiovascular biomarkers are molecules that are produced into the bloodstream when the heart is wounded or under stress. These biomarkers provide essential information regarding the presence of cardiac illness as well as the severity of the condition [3].

Interleukin 6 (IL-6) is an important biomarker for cardiovascular diseases because of its function in inflammation, atherosclerosis, and the inflammatory response. In both stable and unstable coronary disease, high concentrations of IL-6 can predict heart problems, showing that it is a useful biomarker for unfavorable cardiovascular outcomes [3].

In addition to being a cardiovascular bioactive hormone, C-type natriuretic peptide (C-NP) is a member of the natriuretic peptide family. The biological function of C-NP has been investigated in the past, and outcomes have shown that it plays a significant role in the regulation of blood pressure and the maintenance of vascular homeostasis [4,5]. The level of plasma C-NP has been shown to be altered in patients who are suffering from heart failure, according to recent clinical research [6,7].

Because it has the potential to predict cardiovascular risk on a worldwide scale, C-Reactive protein (CRP) has garnered a substantial amount of interest from the scientific community. The change in CRP levels can be attributed to a variety of cardiac conditions, including acute inflammatory disease, peripheral vascular disease, and myocardial infarction [8]. CRP levels of 1.0 mg L^−1^ are sufficient to raise the risk of cardiovascular disease, according to the Centers for Disease Control and Prevention (CDC), according to the effects that have been described [9].

A number of techniques were reported for the determination of the cardiovascular biomarkers, such as flow cytometry [10], colorimetric analysis [11], enzyme-linked immunosorbent assay [12], polymerase chain reaction [13], and chemiluminescence immunoassay [14]. On the other hand, these methods need processing of samples before analysis and therefore, they are time-consuming, making them difficult to employ for on-site and real-time detection. Electrochemical sensors enable rapid responses, high sensitivity, and ongoing monitoring by converting chemical reactions into measurable electrical signals [15,16].

In the area of bioanalysis, it has been demonstrated that practical nanomaterials are desirable choices for use in the construction of sensors for the detection and measurement of bioanalytes: their utilization is facilitating an increase in the surface of the active area, facilitating an increase in the sensitivity of the sensor. The exceptional characteristics of nanomaterials that are derived from phthalocyanines derivatives guarantee that the sensors will have improved performance in terms of sensitivity, selectivity, detection limit, reaction time, and the capacity to multiplex [17].

According to the fact that silver nanoparticles (AgNPs) are commonly recognized for their exceptional optoelectronic and physicochemical capabilities, they have garnered a significant amount of interest in the field of sensor technology [18]. Due to the exceptional electrical conductivity and catalytic activity that AgNPs possess, they have been utilized widely in electrochemical sensors [19].

Three distinct types of phthalocyanines, namely phthalocyanine, cobalt (II) phthalocyanine, and zinc phthalocyanine, were used for the design of the stochastic sensors. These phthalocyanines were chosen as modifiers due to their capacity to supply stable channels, which are essential for stochastic measurements.

As a means of enhancing the electrochemical characteristics of the proposed matrices, carbon nanopowder (CNP) has been enhanced with silver nanoparticles. The innovative aspect of this study is the use of three distinct kinds of porphyrins as modifiers. Three stochastic sensors were developed and tested for the presence of C-NP, CRP, and IL-6 in whole blood samples from patients with cardiovascular illnesses. The concept behind these sensors was to physically immobilize phthalocyanine complexes in carbon paste matrices adorned with AgNPs.

The paper proposed three stochastic sensors designed using the materials described above, as tools for screening tests of whole blood for early and fast diagnosis of cardiovascular diseases.

## 2. Materials and Methods

Analytical-grade carbon nanopowder, AgNPs, phthalocyanine (H_2_Pc, tetrabenzo[b,g,l,q]-5,10,15,20-tetraazaporphyrin), cobalt (II) phthalocyanine, and zinc phthalocyanine were acquired. Monosodium and disodium phosphate, C-NP, CRP, and IL-6 were acquired from Sigma Aldrich in Milwaukee (WI, USA). Fluka was the supplier of the paraffin oil.

The calibration of the sensor was carried out by employing solutions that were prepared through the serial dilution method and buffered in phosphate buffer solution (PBS) with a pH of 7.4. The concentrations of these solutions included 1.00 × 10^−4^–1.00 × 10^−21^ g mL^−1^ for C-NP, 1.00 × 10^−6^–4.00 × 10^−21^ g mL^−1^ for IL-6, and 1.00 × 10^−4^–1.00 × 10^−21^ g mL^−1^ for CRP. Sodium phosphate monobasic monohydrate and sodium phosphate dibasic heptahydrate were combined in order to create PBS. A pH of 7.4 was reached by combining the two solutions in different proportions until the desired result was reached. A sodium hydroxide solution or hydrochloric acid with a concentration of 0.1 mol L^−1^ was used to alter the pH.

The preparation of the solutions involved the utilization of deionized water obtained from a Direct-Q 3 Water Purification System located in Molsheim, France. In order to make the necessary modifications to the pH, a Mettler Toledo pH meter was utilized. During the course of the analysis, room temperature was maintained.

A laptop computer running the PS Trace application version 5.12 was used in conjunction with an EmStat4S potentiostat manufactured by PalmSens BV in Houten, The Netherlands. This allowed for the electrochemical investigations to be carried out. The stochastic microsensors that were proposed were utilized as the working electrode, while a platinum wire was employed as the counter electrode. Additionally, an Ag/AgCl was utilized as the reference electrode, with 0.1 mol L^−1^ KCl serving as the reference electrode. These components composed the electrochemical system. Inspect S, a scanning electron microscope (SEM) manufactured by FEI Company in the Eindhoven, Netherlands, was utilized in order to investigate the surface morphology of the pastes. Using a spot value of 3 and a high voltage (HV) of 30 kV, the samples were analyzed with the LFD detector in low vacuum mode. This was done in order to produce a high picture resolution.

**Figure 1:**
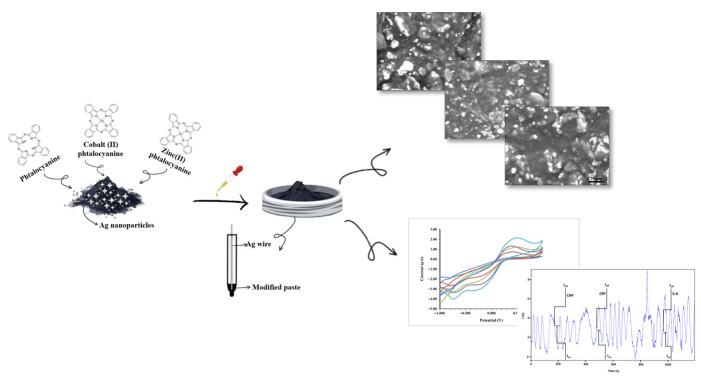
The schematic representation of the design of the stochastic sensors.

The sensors were made by combining 50 mg of AgNPs and 100 mg of carbon nanopowder with paraffin oil to create a uniform paste. This paste was separated into three sections, and each section was chemically modified using 50 μL solutions of 1.0 × 10^−3^ M (in tetrahydrofuran) phthalocyanine, cobalt (II) phthalocyanine, and zinc (II) phthalocyanine. The sensors’ design is shown in Figure 1. A silver wire was used as an electrical contact, and each modified paste was put into a non-conducting plastic tube with an interior diameter of 30 µm. The sensors were denoted as follows: Ph-AgNPs/C nanopowder (Ph-AgCNP), Co(II)Ph-AgNPs/C nanopowder (Co(II)Ph-AgCNP), and Zn(II)Ph-AgNPs/C nanopowder (Zn(II)Ph-AgCNP). Prior to usage, alumina paper was used to polish each sensor’s active surface. After every measurement, deionized water was used to clean the active surface, and paper tissues were used to dry it. The times they were not being used, the sensors were kept at room temperature in a dark place.

### 2.1. Stochastic Method

In order to determine the t_on_ and t_off_ parameters that are required for stochastic analysis, chronoamperometry was utilized. A constant potential of 125 mV vs. Ag/AgCl was chosen, after the potential at which the current should be measured was optimized. It was determined that the signatures of the analytes (the t_off_ parameter) can be easily identified and determined at this potential, which led to the selection of this particular value. On the basis of their signatures, the three biomarkers were detected in the diagrams that were acquired, and the concentration of the biomarkers was determined by utilizing the values of the t_on_ parameter (which were read in between two t_off_’s). Using the calibration equation, analysis was performed to assess the levels of the biomarkers present in the samples. The equation is as follows: 1/t_on_ = a + b × C_biomarker_, where a represents the intercept, b represents the slope (or sensitivity), and C_biomarker_ represents the unknown concentration of the biomarker involved. The signature of the analyte is the generic term for the t_off_ parameter which is often used. The length of time that it takes for the analyte to enter the pore and start blocking the passage of current through it is indicated by this value. On the other hand, this characteristic is directly connected to the molecular identification of the analyte, and it is applied in qualitative analysis. Due to the fact that it is influenced by certain aspects, such as the size, shape, and unfolding capacity of the analyte, it is distinct for each and every analyte’s characteristic. Biological sample analysis took place over the course of 1200 s, whilst solution analysis including solutions containing different concentrations of biomarkers occurred over the course of 360 s.

### 2.2. Samples

The whole blood samples were obtained from patients confirmed to have cardiovascular illnesses in tubes containing EDTA and evaluated right after collection to assess the performance of the stochastic sensors that were proposed. The samples were selected from patients confirmed to have cardiovascular diseases for which concentrations of the biomarkers C-NP, CRP, and IL-6 were relevant for cardiovascular illnesses. The patients selected did not have confirmed inflammations in any part of the body. The Ethics Committee of the University of Medicine and Pharmacy “Carol Davila” Bucharest granted the approval number 3505/9 February 2024. Every participant in the study provided their informed consent. Ten biological samples were analyzed in their original, unmodified state, immediately from the hospital, without prior processing of the entire blood sample. The proposed stochastic sensors were employed for all validation assessments. For every whole blood sample, ten diagrams were recorded.

## 3. Results

### 3.1. Morphology of the Active Surface of the Stochastic Sensors

**Figure 2:**
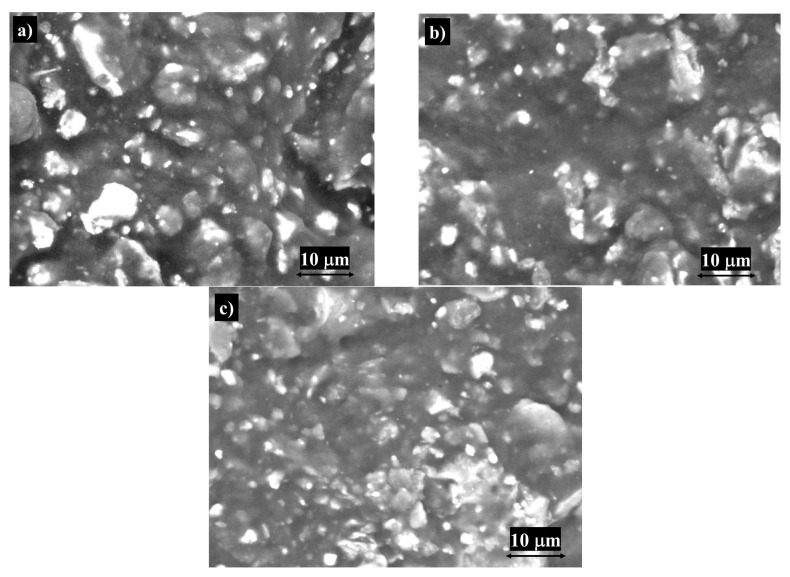
SEM analysis for the three proposed stochastic sensors used for the assay of C-NP, IL-6 and CRP: (**a**) Ph-AgNPs/CNP, (**b**) Co(II)Ph-AgNPs/CNP, and (**c**) Zn(II)Ph-AgNPs/CNP.

The SEM technique was utilized in order to investigate the surface morphologies of the sensors (shown in Figure 2). The SEM micrographs reveal a heterogeneous and porous carbon-based matrix, which provides a high surface area for further functionalization. Silver nanoparticles are observed as bright, well-dispersed spherical features, confirming their successful incorporation into the paste. The presence of the different types of phthalocyanines (Figure 2a–c) leads to a more uniform coating and enhances the surface roughness, which is expected to improve electron transfer and catalytic activity. The overall morphology demonstrates the synergistic integration of carbon, silver nanoparticles, and different types of phthalocyanines, providing a conductive and catalytically active surface suitable for electrochemical applications. The presence of the necessary channels for stochastic sensing can be seen in Figure 2a.

### 3.2. Electrochemical Characterization of the Stochastic Sensors

To conduct an analysis and evaluation of the performance of the three modified sensors in addition to the performance of the sensors that were not modified, cyclic voltammetry (CV) and electrochemical impedance spectroscopy (EIS) were utilized. In order to conduct the CV measurements, carbon nanopowder (CNP), silver–carbon nanopowder (AgCNP), Ph-AgCNP, Co(II)Ph-AgCNP, and ZnPh-AgCNP were utilized as the working electrodes. Experiments were conducted in a solution that contained a mixture of potassium ferrocyanide (K_3_[Fe(CN)_6_]) and potassium chloride (KCl) at a concentration of 0.1 M. The experimental potentials ranged from −1.0 V to 1.0 V.

**Figure 3:**
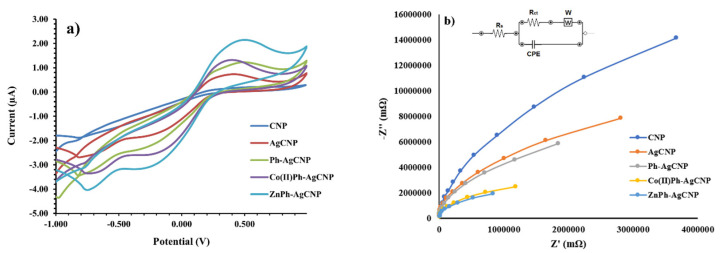
(**a**) Cyclic voltamograms of current versus potential (working conditions: potential 0.025 V; scan rate 0.1 V s^−1^) in a 5.0 × 10^−3^ M K_3_[Fe(CN)_6_] (0.1 M KCl) solution using CNP (blue line), AgCNP (red line), Ph-AgCNP (green line), Co(II)Ph-AgCNP (purple line), and ZnPh-AgCNP (light blue line). (**b**) Electrochemical impedance spectra recorded for CNP (blue line), AgCNP (orange line), Ph-AgCNP (grey line), Co(II)Ph-AgCNP (yellow line), and ZnPh-AgCNP (light blue line).

After the simple carbon paste has been treated with the three different forms of phthalocyanines, a significant change in the electrochemical behavior can be observed. As can be observed from the curve in Figure 3a, the bare sensor does not exhibit any redox peaks. Following the addition of the AgNPs, there is a small but noticeable rise in the peaks. The anodic peak current (Ipa) and cathodic peak current (Ipc) were measured to be 0.78 µA and −0.46 µA, respectively. Following the addition of Ph to the AgCNP electrode, the responsive redox peak currents increase, with Ipa being equal to 0.85 µA and Ipc being equal to −0.55 µA. After the addition of Co(II)Ph to the AgCNP electrode, the response redox peak currents exhibit a significant increase (Ipa = 1.38 µA; Ipc = −0.87 µA). One of the most pronounced redox peaks was revealed by the ZnPh-AgCNP sensor, which was characterized by an anodic peak current (Ipa) of 1.57 µA and a cathodic peak current (Ipc) of −1.05 µA. This is due to the fact that Zn(II)Ph possesses a strong electrical conductivity and a large surface area. Therefore, we are able to draw the conclusion that all three sensors were successfully modified, indicating that there was an improvement in the electrochemical signal.

A mixed solution consisting of 5 mM [Fe(CN)_6_]^3−/4−^ and 0.1 M KCl was used to conduct the analysis of the Nyquist graphs of the modified stochastic electrodes. The frequency range that was utilized for the analysis was between 0.1 Hz and 100 kHz. As can be seen in Figure 3b, the results are displayed. The Nyquist plot displays a semicircular portion at high frequencies, while a linear part is displayed at low frequencies. This is where the semicircular section is found. There is a strong connection between these sections and the diffusion-controlled process and the electron transfer-limited process, respectively. The dimension of a semicircle is typically used to indicate the electron charge transfer resistance value, which is denoted by the symbol R_ct_. For the purpose of determining the values of R_ct_, the Randles circuit, which is depicted in the inset of Figure 3b, was utilized. Within the context of this circuit, the symbol W represents the Warburg impedance, Rs is the resistance of the electrolyte, and CPE is the indication of the constant phase element. In the low-frequency domain, the sensor based on CNP exhibited a significant semicircle that was well defined, as shown in Figure 3b. This semicircle is suggestive of a high electrical resistance, which is represented by the value R_ct_ = 5.42 × 10^7^ Ω. After the modification of the sensor based on CNP with AgNPs, the diameter of the semicircle decreased, as indicated by the value of R_ct_, which was 2.37 × 10^7^ Ω. Within the context of Ph-AgCNP, it is seen that a quasi-semicircle with an even smaller radius (R_ct_ = 1.92 × 10^7^ Ω) is observed. For Co(II)Ph-AgCNP, the obtained radius for the semicircle was R_ct_ = 4.62 × 10^6^ Ω. The smallest R_ct_ value was obtained for the Zn(II)Ph-AgCNP, having an R_ct_ = 1624 Ω, which is correlated with the smallest semicircle. In conclusion, the CNP that had been treated with AgNPs and Zn(II)Ph displayed a diminishing quasi-semicircle and a R_ct_ value that was much lower than the value of the CNP that had not been modified.

### 3.3. Response Characteristics of the Developed Stochastic Sensors

There are two stages involved in the stochastic detection process, and it is dependent on the interactions which occur between the selected compounds and a conducting channel. Under the influence of a constant voltage, an electric current flows across the conductive channel.

The first step is to extract the substance of interest from the solution at the border amid the outer layer of membrane and the solution. When the analyte molecule moves through the channel and blocks it, the magnitude of the current decreases until it reaches zero. When referring to this characteristic of the analyte, the term “t_off_ parameter” is utilized. The analyte undergoes redox reactions and engages in chemical interactions with the channel wall during the second step of the process. “t_on_” is the term used to describe the amount of time that must pass for this process to reach a state of equilibrium. As shown in Table 1, the response characteristics of the stochastic sensors that were developed and employed for the C-NP, IL-6, and CRP assays are shown. Wide linear concentration ranges were achieved for the biomarkers that were assayed, as well as very low limits of quantitation (LOQ) and high sensitivities. Based on the obtained results, it is clear that the suggested sensor offers a method that is both reliable and accurate for simultaneous identification of C-NP, IL-6, and CRP.

For the assay of IL-6 biomarker, very similar results were obtained when the three proposed sensors were used. In terms of LOQ, we have the same magnitude order (10^−21^ g mL^−1^), while the concentration ranges were appropriate as results. The highest sensitivity was obtained for the Zn(II)Ph-AgCNP-based sensor. For CRP, the Co(II)Ph-AgCNP-based sensor had the best sensitivity, with a value of 6.73 × 10^10^ s^−1^ g^−1^ mL, compared with the other two sensors. Instead, the best LOQ values (1.00 × 10^−21^ g mL^−1^) were obtained when Co(II)Ph-AgCNP- and Ph-AgCNP-based sensors were used. In the case of the C-NP biomarker, the best results in terms of sensitivity, with a value of 9.13 × 10^12^ s^−1^ g^−1^ mL, were achieved for the Co(II)Ph-AgCNP-based sensors, and similar results were obtained for the other two sensors. In conclusion, comparing all the results obtained, the sensor of choice was the one based on Zn(II)Ph-AgCNP.

### 3.4. Selectivity of the Stochastic Sensors

The selectivity of the stochastic sensor is established by the variance in the recorded signals (t_off_ values) for IL-6, CRP, and C-NP, in contrast to those acquired for other biomarkers or substances derived from biological samples (Table 2). This distinction has been recognized as the variation between the signatures.

TNF-α, VCAM-1, MCP-1, and LHD were chosen as possible interferences for the determination of IL-6, CRP, and C-NP in whole blood samples.

The results shown in Table 2 prove that there is a difference of minimum 0.2 s between the signatures (t_off_ values) of IL-6, CRP, and C-NP and the signatures of TNF-α, VCAM-1, MCP-1, and LHD. Accordingly, there is no interference of TNF-α, VCAM-1, MCP-1, and LHD in the determination of IL-6, CRP, and C-NP.

### 3.5. Stability Measurements for the Stochastic Sensors Based on Phthalocyanines

Stability tests were done accordingly with the protocol recommended by IUPAC [20]. Ten stochastic sensors of each type were created and stored in accordance with the preceding description. A new stochastic sensor was taken out of storage on a daily basis and immersed in each of the solutions that contained IL-6, CRP, and C-NP at different concentrations. Furthermore, the degree of sensitivity achieved by each measurement were recorded for the purpose of assessment following each stochastic sensor batch that had been used up in thirty days. The end-of-period data proved that the stochastic sensors were quite stable throughout time, as the variation in sensitivities over time was less than 0.10%, despite the different modifiers (Ph, Co(II)Ph, or Zn(II)Ph) employed in the design of the stochastic sensors.

### 3.6. Simultaneous Determination of the Selected Biomarkers in Biological Samples

**Figure 4:**
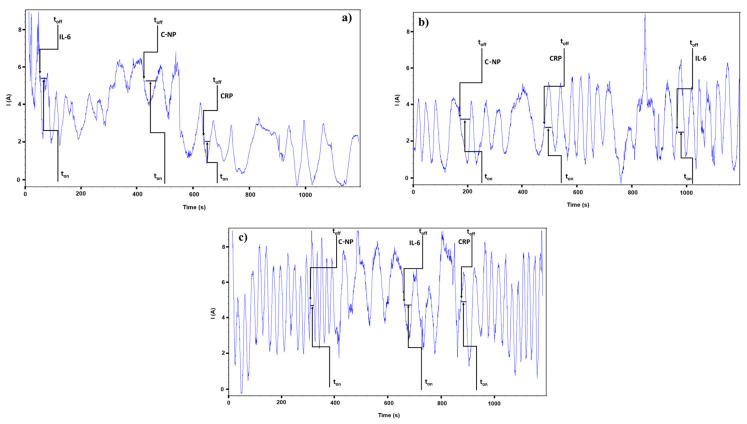
Determination of IL-6, CRP, and C-NP in whole blood samples using the stochastic sensors (**a**) Co(II)Ph-AgNPs/CNP, (**b**) Ph-AgNPs/CNP, and (**c**) Zn(II)Ph-AgNPs/CNP.

Stochastic sensors were utilized to conduct an analysis on a total of ten whole blood samples that were collected from individuals who had received a diagnosis of cardiovascular illnesses. Pattern recognition was the first phase, and its primary objective was to determine the distinctive t_off_ values (signatures) that are connected with C-NP, IL-6, and CRP in the diagrams that were obtained by employing the Ph-AgCNP, Co(II)Ph-AgCNP, and Zn(II)Ph-AgCNP stochastic sensors (Figure 4).

After the t_off_ values were read, the corresponding t_on_ values were read between two t_off_ values. By employing the stochastic method described previously, the ton values were utilized in order to determine the amounts of C-NP, IL-6, and CRP that were present in whole blood samples. The results of the determination of the biological samples are presented in Table 3.

The data obtained from the three stochastic sensors and the ELISA were found to have very strong correlations with one another (Table 3). With a confidence level of 99% (the theoretical t-value is 4.03), an analysis of the data using Student’s paired *t*-test for each biomarker was conducted. All of the t-values that were calculated were lower than 3.00, which is smaller than the value that was predicted by theory. There is no statistically significant difference between the data that was acquired, as demonstrated by the findings that were obtained using the stochastic sensors (Table 3). As a result, it can be concluded that the stochastic sensors are capable of performing an efficient assessment of C-NP, IL-6, and CRP in the selected biological samples at the same time.

### 3.7. Recovery Tests of IL-6, CRP, and C-NP in Whole Blood

The recovery testing of IL-6, CRP, and C-NP in whole blood samples was carried out with the use of the Ph-AgCNP-, Co(II)Ph-AgCNP-, and Zn(II)Ph-AgCNP-based stochastic sensors. Following the determination of the initial concentrations of IL-6, CRP, and C-NP in whole blood samples, a series of different concentrations, ranging from extremely low to extremely high levels (determined to be within the operational concentration range of each sensor), were incorporated into the samples, and subsequent assessments were conducted. An analysis was carried out to determine the difference between the quantity that was introduced and the quantity that was added of IL-6, CRP, and C-NP that was found in whole blood samples. Table 4 contains the results that were obtained.

The results of the assays for C-NP, CRP, and IL-6 were extremely high—above 99.10%. Overall, the values for RSD were below 0.10% (Table 4). Using the Ph-AgCNP, Co(II)Ph-AgCNP, and Zn(II)Ph-AgCNP stochastic sensors, one can draw the conclusion that IL-6, CRP, and C-NP are possible to ascertain with great precision and accuracy from whole blood samples. The results of the recovery test and the paired *t*-test support this conclusion.

## 4. Discussions

The response characteristics of the stochastic sensors made them suitable to be used in screening tests of whole blood for IL-6, CRP, and C-NP. High sensitivities and very low limits of determination, as well as a wide working concentration range, can facilitate determination of IL-6, CRP, and C-NP in whole blood samples of healthy patients as well as in the whole blood samples of patients with CVDs. To our knowledge, this is the only method able to simultaneously determine the three biomarkers simultaneously. Also, the proposed stochastic sensors proved to be, accordingly with the results shown in Table 3 and Table 4, suitable screening tools for IL-6, CRP, and C-NP in whole blood. There is good agreement between results obtained with the proposed sensors and the results obtained using the standard method—ELISA, demonstrated by the Student *t*-test. The suitability of utilization of these stochastics sensors as screening tools was also proved by the recovery tests, when recoveries higher than 99.10% were recorded for the three biomarkers. Although—accordingly with the response characteristics of the proposed stochastic sensors—the sensor of choice is the one based on Zn(II)Ph-AgCNP, all three sensors may be successfully used for the screening of whole blood for fast and early diagnosis of cardiovascular diseases.

Comparing the screening method that uses the proposed stochastic sensors with the methods reported earlier [11,12,13,14], those that have been successfully used for the analysis of samples for these biomarkers in specialized clinical laboratories, the proposed method using the stochastic sensors is faster and can be used for on-site analysis, does not require any sample processing, can be used on a wide concentration range, and is more accurate and reliable because the sample is analyzed as it is picked up from the patients. Accordingly, a screening test using one of the proposed sensors as a screening tool can be a good alternative for ELISA in clinical analysis.

## 5. Conclusions

In this study, three different stochastic sensors were employed to measure three different biomarkers—C-NP, CRP, and IL-6—in whole blood samples. The sensors were based on CNP and silver nanoparticles that had been modified with three different forms of phthalocyanines. When applied to biological samples taken from patients suffering from cardiovascular diseases, the suggested stochastic sensors reliably identified patterns indicating the presence of cardiovascular biomarkers. The primary function of these sensors is to be used as screening tools for whole blood samples in order to achieve fast and early diagnosis of cardiovascular diseases, or to find the state of health of the patients who already have a diagnosis of cardiovascular disease.

## Figures and Tables

**Table 1:** Response characteristics of the three proposed sensors for IL-6, CRP, and C-NP analysis (N = 10).

Sensor Based on AgNP/CNP and	Biomarker	t_off_ (s)	Calibration Equation; Correlation Coefficient (r)	Sensitivity (s^−1^ g^−1^ mL)	LOQ (g mL^−1^)	Working Concentration Range (g mL^−1^)
**Ph**	IL-6	1.1	1/t_on_ = 0.46 (±0.02) + 3.04 × 10^9^ × C r = 0.9966	3.04 (±0.03) × 10^9^	1.00 × 10^−21^	1.00 × 10^−21^–1.00 × 10^−6^
	CRP	1.7	1/t_on_ = 0.05 (±0.01) + 6.48 × 10^9^ × C r = 0.9995	6.48 (±0.03) × 10^9^	1.00 × 10^−21^	1.00 × 10^−21^–1.00 × 10^−4^
	C-NP	0.8	1/t_on_ = 0.45 (±0.02) + 2.05 × 10^10^ × C r = 0.9999	2.05 (±0.03) × 10^10^	1.00 × 10^−21^	1.00 × 10^−21^–1.00 × 10^−4^
**Co(II)Ph**	IL-6	0.5	1/t_on_ = 0.53 (±0.05) + 9.17 × 10^8^ × C r = 0.9999	9.17 (±0.02) × 10^8^	4.10 × 10^−21^	4.10 × 10^−21^–1.00 × 10^−6^
	CRP	1.4	1/t_on_ = 0.16 (±0.04) + 6.73 × 10^10^ × C r = 0.9999	6.73 (±0.03) × 10^10^	1.00 × 10^−21^	1.00 × 10^−21^–1.00 × 10^−6^
	C-NP	0.8	1/t_on_ = 0.52 (±0.08) + 9.13 × 10^12^ × C r = 0.9995	9.13 (±0.03) × 1012	1.00 × 10^−21^	1.00 × 10^−21^–1.00 × 10^−4^
**Zn(II)Ph**	IL-6	1.0	1/t_on_ = 0.2 (±0.05) + 1.13 × 10^9^ × C r = 0.9999	1.13 (±0.04) × 10^9^	6.40 × 10^−21^	6.40 × 10^−21^–1.00 × 10^−6^
	CRP	0.8	1/t_on_ = 0.11 (±0.08) + 7.98 × 10^9^ × C r = 0.9993	7.98 (±0.07) × 10^9^	1.00 × 10^−15^	1.00 × 10^−15^–1.00 × 10^−6^
	C-NP	2.0	1/t_on_ = 0.13 (±0.02) + 3.73 × 10^9^ × C r = 0.9999	3.73 (±0.02) × 10^9^	1.00 × 10^−20^	1.00 × 10^−20^–1.00 × 10^−6^

**Table 2:** Selectivity of the stochastic sensors for IL-6, CRP, and C-NP determination.

Sensor Based on AgNP/CNP	Biomarker	t_off_ (s)
**Ph**	IL-6	1.1
	CRP	1.7
	C-NP	0.8
	TNF-α	0.3
	VCAM-1	1.5
	MCP-1	2.0
	LHD	3.2
**Co(II)Ph**	IL-6	0.5
	CRP	1.4
	C-NP	0.8
	TNF-α	1.7
	VCAM-1	2.2
	MCP-1	2.6
	LHD	3.5
**Zn(II)Ph**	IL-6	1.0
	CRP	0.8
	C-NP	2.0
	TNF-α	0.3
	VCAM-1	2.5
	MCP-1	1.5
	LHD	3.0

**Table 3:** Determination of C-NP, IL-6, and CRP in various whole blood samples using the Co(II)Ph-AgNPs/CNP, Ph-AgNPs/CNP, and Zn(II)Ph-AgNPs/CNP stochastic sensors (N = 10).

Sample No.	Microsensor Based on AgNPs/CNP	IL-6 (ng mL^−1^)		CRP (ng mL^−1^)		C-NP (pg mL^−1^)	
		Stochastic Method	ELISA	Stochastic Method	ELISA	Stochastic Method	ELISA
**1**	Ph	63.48 ± 0.05	64.00 ± 0.17	19.67 ± 0.03	19.15 ± 0.19	395.25 ± 0.04	395.40 ± 0.24
	Co(II)Ph	63.84 ± 0.02		19.78 ± 0.02		398.94 ± 0.02	
	Zn(II)Ph	63.66 ± 0.01		19.23 ± 0.04		398.19 ± 0.03	
**2**	Ph	110.15 ± 0.03	109.50 ± 0.2	9.39 ± 0.03	9.00 ± 0.20	23.00 ± 0.03	22.00 ±0.41
	Co(II)Ph	110.00 ± 0.04		9.50 ± 0.02		22.90 ± 0.02	
	Zn(II)Ph	110.75 ± 0.03		10.00 ± 0.04		22.87 ± 0.02	
**3**	Ph	121.90 ± 0.04	121.97 ± 0.40	7.58 ± 0.03	7.12 ± 0.15	529.10 ± 0.03	525.40 ± 0.20
	Co(II)Ph	122.15 ± 0.03		7.51 ± 0.03		527.39 ± 0.04	
	Zn(II)Ph	122.39 ± 0.02		7.79 ± 0.04		528.53 ± 0.04	
**4**	Ph	116.16 ± 0.03	115.50 ± 0.30	6.20 ± 0.02	6.00 ± 0.10	20.36 ± 0.03	20.40 ± 0.14
	Co(II)Ph	116.30 ± 0.02		6.14 ± 0.03		21.02 ± 0.03	
	Zn(II)Ph	115.97 ± 0.03		6.90 ± 0.01		20.95 ± 0.02	
**5**	Ph	133.23 ± 0.04	130.90 ± 0.20	3.68 ± 0.03	3.00 ± 0.28	20.30 ± 0.02	20.50 ± 0.15
	Co(II)Ph	131.09 ± 0.02		3.40 ± 0.02		20.83 ± 0.01	
	Zn(II)Ph	131.14 ± 0.05		3.18 ± 0.02		21.17 ± 0.03	
**6**	Ph	106.02 ± 0.03	105.40 ± 0.12	23.15 ± 0.01	24.40 ± 0.30	8.50 ± 0.01	8.50 ± 0.21
	Co(II)Ph	106.16 ± 0.02		24.04 ± 0.03		8.47 ± 0.03	
	Zn(II)Ph	105.80 ± 0.02		23.90 ± 0.02		8.53 ± 0.02	
**7**	Ph	137.93 ± 0.01	135.20 ± 0.15	4.63 ± 0.03	4.60 ± 0.20	292.90 ± 0.04	294.10 ± 0.10
	Co(II)Ph	135.15 ± 0.02		4.63 ± 0.02		293.57 ± 0.03	
	Zn(II)Ph	135.11 ± 0.02		4.81 ± 0.03		292.97 ± 0.02	
**8**	Ph	130.76 ± 0.04	129.80 ± 0.20	5.12 ± 0.03	5.20 ± 0.15	20.39 ± 0.03	20.45 ± 0.15
	Co(II)Ph	129.95 ± 0.03		5.12 ± 0.04		21.16 ± 0.03	
	Zn(II)Ph	130.40 ± 0.02		5.40 ± 0.02		21.40 ± 0.02	
**9**	Ph	545.90 ± 0.02	545.10 ± 0.25	11.65 ± 0.03	11.50 ± 0.10	11.15 ± 0.03	11.20 ± 0.25
	Co(II)Ph	546.11 ± 0.03		11.30 ± 0.03		11.20 ± 0.05	
	Zn(II)Ph	545.87 ± 0.04		11.48 ± 0.02		11.70 ± 0.03	
**10**	Ph	130.76 ± 0.02	130.50 ± 0.42	13.35 ± 0.04	13.50 ± 0.24	502.69 ± 0.02	501.80 ± 0.32
	Co(II)Ph	131.12 ± 0.02		13.70 ± 0.02		501.95 ± 0.04	
	Zn(II)Ph	130.48 ± 0.02		13.29 ± 0.03		502.14 ± 0.03	

**Table 4:** Recovery of IL-6, CRP, and C-NP using the proposed stochastic sensors.

Sensor Based on AgNPs/CNP and	%, Recovery IL-6	%, Recovery CRP	%, Recovery C-NP
**Ph**	99.23 ± 0.02	99.12 ± 0.04	99.65 ± 0.02
**Co(II)Ph**	99.45 ± 0.04	99.30 ± 0.03	99.60 ± 0.03
**Zn(II)Ph**	99.78 ± 0.04	99.55 ± 0.02	99.90 ± 0.02

## Data Availability

Data is unavailable due to ethical restrictions.
